# Nervous Agency in Inflammatory Action

**Published:** 1860-10

**Authors:** L. S. Ellis

**Affiliations:** Chicago, Ill.


ARTICLE 3.

### NERVOUS AGENCY IN INFLAMMATORY ACTION.

BY L. S. ELLIS, A. M., M. D., CHICAGO, ILL.

It may not be my privilege to present anything new, unheard, or unthought of, on the above topic; but I hope to present existing knowledge and hitherto expressed opinions, scattered through more modern medical literature, so as to make them bear more directly on therapeutic measures and curative appliances. Thereby a sound and immovable basis will be found for principles in medical treatment which are fast gaining the ascendency at the present time. Greater revolutions transpire neither in the moral or political world, than constantly and almost imperceptibly occur in the medical profession. There is no principle so well founded as not to bear and require an occasional review in the accumulated light and labors of shining and laborious men in our ranks. Ovservers multiply in every department; the storehouse is rich in their accumulations; gleaners and gleanings are abundant, and minds are not wanting who comprehend all these, and will, out of all this apparently confused mass, bring out a system so in accordance with nature's laws, as that our ministrations for her relief shall be more and more in harmony therewith. Dr. Todd has well said, "It will not be affirmed by any one, that the doctrine of a science so abstruse and so difficult as pathology, should not be reviewed and reconsidered from time to time. There never was a period when the candid and ample reconsideration of general pathology, promised more fruitful results than at the present time. Our vastly extended acquaintance with anatomy and physiology, the greatly enlarged security of the basis on which the knowledge of function rests, the much increased accumulation of facts of clinical history, all afford the most important data for new inductions." Actuated as he was by such comprehensive views, we lament that his light, so brilliant, has so soon gone out, and labors so enthusiastic have so soon terminated. The light of life has indeed gone out. The light of his mind shines with no diminished lustre, and contributes to establish wiser physiological and pathological principles and more successful remedial appliances.

My earliest lessons learned about inflammation embraced the question duly considered, as to whether imflammatory action consisted in increased or diminished action in the vascular system, and as determined, blood-letting took its place among general principles as a stimulant or sedative. "Sthenic or asthenic" were as household words, and doubtless have been, and are, words of mighty import in the minds of many of the teachers and the taught of our profession. Information acquired from the clinical experience of others, observations in practical life, and more extended research, carried the conviction that the "*Fons et origo*" of inflammation must be sought behind any increased or diminished vascular action. The first visible change in muscular contraction, is a shortening of the muscular fibre, but the real primary change is in the nervous filaments associated with it. The nervous system has an immediate influence upon all the molecular changes occurring in the system, whether of nutrition, secretion, &c., or their abnormal action, constituting inflammation, or disease in any of its protean shapes. I shall assume, and endeavor to prove, that *nervous irritation is the primary change in the pathology of inflammation*, and that all consequent changes occurring in the vascular system or other structures, are but sequences, and that we must seek for the relation of disease and treatment in this primary nervous change. In support of the foregoing proposition I shall adopt the following order in argument:

First—The Anatomy and Physiology of the ultimate nerve fibres, and of the capillary system.

Second—Microscopic Appearances in Inflammatory Action.

Third—Effect of Irritation at the centre or along the course of the nerves.

Fourth—The Mode of Action of Remedial Measures.

Fifth—Opinions of Authors and Teachers.

Sixth—Practical Inferences.

It may not be amiss to contemplate the nervous system, embracing the brain, spinal cord, ganglia, and filaments, as the "neuro skeleton." The other structures, such as bones, muscles, blood-vessels, &c., stand as supports, instruments, and ministers to its wants and destiny. All other functions are but subordinate to the controlling influence of the nervous power. A portion of the nervous system, ever vigilant in duty exerts its influence in the growth of tissue, and does its part in maintaining the integrity of the whole from neverceasing waste. Other filaments stand as myriads of sentinels on the outposts, to guard the body as the mind's instrument. Others, sti'l, as conveyors of special sensation, educate the mind for a higher sphere and a noble destiny.

The anatomy of the ultimate nerve fibre is still involved in no little obscurity. Dr. Draper says that "the nerve fibres terminate in various ways. Their ends may thin out and become free, or they may form a loop and so return back in their course."—Physiology, p. 263.

It is not unlikely that the nervous distribution to the capillary system is accomplished by one or the other of the means mentioned by Prof. Draper. The hindrance from that obscurity regarding their anatomical structure, is overcome in a great measure by physiological research, and the phenomena of nutrition, secretion, &c., prevent a necessity for the presence of nerves, for their influence in these molecular changes, ever varying from impressions on the nervous system. "It can scarcely be doubted, that it is by some influence exercised over the molecular actions, to which the blood is subject in the capillaries, that the nervous system can operate on the functions of nutrition, secretion, &c., and this influence may not be improperly termed *vital*, if, by so designating it, we merely imply that its nature and mode of operation are unknown, but that it is closely connected with those actions which are altogether peculiar to living beings. All the facts which bear upon the question of the connection between nervous agency and the forces maintaining the capillary circulation, have an equal relation to the functions of nutrition and secretion in general; and as already shown the nervous system also influences these by the control it exerts over the diameter of the blood-vessels."—Carpenters Phys.

While thus we have an influence exerted over the phenomena of nutrition and secretion in their normal operations, we have a like influence, which, if disturbed, will exert a power to disturb and modify nutrition and secretion. This influence is sometimes exerted in exciting, sometimes checking and modifying these functions. Thus every one is familiar with he effects upon the vascular system of severe concussion of the brain. So with mental impressions—nervous anxiety has a marked influence over these functions.

So delicate and complete is the arrangement of the capillary circulation on the surface, that the prick of the finest needle wounds several of these vessels, and also causes pain, showing that the distribution of nerves is equally minute and complete and that nerve fibrils stand as sentinels to guard each minutest point. Each papilla has its numerous nerves, not one is destitute. Each muscular fibre has its accompanying nervous filament, acting simultaneously on each muscle cell. Cruvielhier says that nerves act on muscular fibre by contact, and not through a nervous atmosphere, as claimed by Dr. Reil. Thus, by reviewing all the tissues, we would find a sufficiently ample distribution of nerves to take their place and exert their influence in the phenomena of inflammation.

The microscopic appearances in inflammatory action are familiar to each member of our profession, but it may not be amiss to mention some of the leading characteristics, as a connecting link in the argument. The first effect of an irritant upon the smaller vessels of the part is comparatively slight, but important in its bearing. It may cause a slight contraction or expansion of the walls of the capillary vessels. At the same time the blood slackens or even retrogrades, as if it had received some shock through a medium between the irritant and itself. Very soon the vessels dilate, and the blood, as if recovered from its temporary shock renews its course with increased velocity. Shortly afterward the blood slackens in its course again, and this may continue till complete stagnation occurs. Here the phenomena may end and the blood resumed its course, and the vessels their normal calibre, or all the further results may follow in their usual course. Other changes in the constituent elements of the blood may recur during this time, but they are not of importance in the examination now before us. They all occur as sequences or as results of earlier changes. These changes, no doubt, follow the abnormal nervous influence which first produced the change in the blood vessels and the movements of their contents. We have seen that a distribution of nerves, universal in regard to the body, is proved by anatomy and physiology, and here, in inflammation, we have an influence primary, and continued so long as the irritant continues; we have local shock upon the blood in the capillaries, parallel to the universal shock in concussion, while all the subsequent changes are directed so as to accomplish the removal of the irritant to the nerves of the part.

It is proper next to notice the effect, at the periphery, of irritation at the center, or along the course of the nerve. In all instances this is followed by increased action in the capillary system, and when a gland is involved, by increased activity in its functions. By the division of the nerves distributed to any part, inflammation, with all its train of symptoms and results, follows in rapid and sure succession.

M. Claude Bernard says that by exciting the solar pexus and its efferent branches, both diarrhœa and dysentery occur, and all the habitual anatomical lesions are present; acute peritonitis has been induced, with all its consequences. Fever itself, that essentially medical symptom, can be excited by mere mechanical irritation of the nervous system. Pleuritis, with purulent deposit, may be induced in an animal previously enfeebled, by division of the great sympathetic nerve.

"It is therefore, a fact that the perverted state of the nervous system, gives rise to a great variety of diseases, not only of a general, but also of a local character; deprive a muscle or a bone of its nervous supply, and you will have as a consequence, fatty degeneration in the one case, and rickets in the other; in fact, if you tie the nerves which enter the nutritive foramina of a bone, you will very soon see the cells of the lamellar structure increase in size, the vessels become more numerous, and all the phenomena of rickets follow in rapid succession."—M. Claude Bernard's Lectures.

In experiments on the moter nerve of the parotid gland this same distinguished observer found that by galvanization of its peripheric extremity after it had been divided, an abundant secretion takes place from the gland, and the venous blood becomes a bright red, a phenomena always observed when the gland is in state of activity.

"Division of the fifth pair of nerves within the cranium, or of its ophthalmic branch, is followed by inflammation of the corresponding eye, which usually goes on to complete and permanent destruction of the eye. Immediately after the operation the pupil becomes contracted and the conjunctiva becomes insensible. The inflammation soon spreads to the iris which becomes covered with a layer of inflammatory exudation, the cornea increases in opacity and becomes ulcerated and the humors discharged. Sometimes the inflammation subsides and sight is restored."—Dalton's Physiology, p. 397.

Sufficient evidence is thus found to indicate clearly that irritation at the center, or along the course of the nerves will produce inflammation at the periphery. In this connection it must be observed that the primary change is in the nerve and that the vascular changes occur only as influenced by the change in the nervous system.

The *modus operandi* of remedial measures becomes an important question in this connection. It is the every-day experience of every practicing physician, that the effect of remedies becomes an important aid in the diagnosis of obscure disease. We may gather much of our knowledge of the character of any epidemic constitution of prevailing disease, by the effect of remedies. So the action of medicines becomes of equally important bearing in any inquiry concerning more general disease. Space forbids a full discussion of the action of remedial agents in inflammation. Bloodletting enters largely into the discussions of our standard authors in the treatment of inflammation, but it is so little practiced in every-day life that it scarcely deserves consideration save in works complete as treatises. Legitimately employed, it becomes an aid to nature's efforts to rid itself of the source of disease. By bloodletting its advocates propose so to change the quantity or quality of the blood as to render it more efficient in its office, or "restore the relation of the vessel and its contents." But in inflammation, waste and repair lose their equilibrium. The nervous influence has lost its power to sustain nutrition, and waste goes on, not only with wonted, but increased activity.

In this light he proceeds with caution who thrusts the lancet for general bloodletting. But the nervous system may be overwhelmed by the accumulated blood, changed in character and quantity. The nerves demand a relief from the burden to give them again an opportunity to resume their control over the vessels. Then bloodletting comes in as a rational adjuvant in the process of restoration.

So in the action of a cathartic on the abdominal viscera. The temporary relief of the conjested parts affords a rallying point for the nerves, and they again resume their office and influence in the multiplied functions of the parts. But these more general considerations must give place to a study of more specific remedies. Among these opium stands foremost. On the question of its action, Headland says: "The operations for which opium is most famed are actions on the powers which are universally attributed to the nervous system. The effects of opium occur immediately on absorption, a suddenness of action found in no medicine that acts on the blood. Opium produces no direct change in the blood, and has no chemical power of initiating such change. We may, therefore, reasonably conclude that opium acts on the nervous system immediately and bodily, without the intervention of the fluids otherwise than that they carry it to the part on which it tends to operate. Opium acts on the nervous system, and this is its primary and chief action. How it exerts its power over nerve, or how nerve medicines in general exert such power, I do not pretend to say. *I conceive that it is by a certain intimate or molecular relation existing between the particles of the agent and the particles of the nerve acted upon.*"

Within a few years past opium has received much attention from observers, and its rank in importance has been greatly advanced. It is now considered a powerful remedial agent, instead of a mere *placebo* to quiet pain, nervousness, &c., while the treatment proper was accomplishing its intended office. This view no longer holds with inquiring minds. Opium stands as the *sine qua non* in the treatment of large and important classes of disease. It has been demonstrated beyond peradventure by Dr. A. Clark of New York that opium alone is sufficient, if timely administered, to control the severest cases of puerperal peritonitis. General peritonitis yields equally surely to its influence. Inflammation of the pleura, in its incipient stage, more especially, yields to the magic influence of opium. I believe that it only wants a clear head and brave heart, to show that it would have a like influence, if timely administered, in inflammations of the meninges of the brain. Every one knows its importance in the treatment of all inflammation or irritation of the mucous membrane. Accepting the explanation of Headland, on its manner of action, we have a clear exposition of the reason of its control over inflammation, as shown above, and we have an unanswerable argument pointing to the nervous system as sustaining the primary lesion.

But before we leave this division of the discussion, we must briefly notice the action of a few other remedies. The popularity of arnica in irregular practice is well known, and its valuable properties are becoming known to the profession, and where known it is extensively used. "We know little of the ultimate action of any medicine or external application, but the probability is that arnica produces anæsthesia of the cutaneous nerves, and exerts some influence on the ganglionic nerves which surround the blood-vessels and regulate their action, for it certainly arrests the formation of thrombus and ecchymosis."—*London Lancet*, Feb., 1860, p. 93.

Dr. J. R. Cushing, in the *Atlanta M. and S. Jour.*, says: "I believe aconite has a direct curative action through its influence on the nervous system, independent of its nauseating properties."

"Dr. Headland says: "It acts especially on the superficial sensory nerves, and tends to extinguish feeling and pain."

Quotations in this connection might be multiplied but the growing proportions of our article warns us to turn to other considerations. All along through our standard works we find intimations pointing towards the conclusions we have endeavored to arrive at. Such a thing may be, say they, but no therapentic measures are based thereon, and hence the intimations are practically valueless. After stating that there is no warrant for assuming that the vital contraction of the capillaries produces the changes observed in inflammation.

Dr. Watson says; "The inquiry might be more properly directed, I think, toward the vital conditions of the *nerves* of the part."

Dr. Wood says, in writing on the theory of inflammation: "An irritant cause acts on a part of the body, either immediately from without, or through the blood, or by the instrumentality of the nerves. Morbidly increased action or irritation is excited in the part, embracing, probably, all its vital constituents as well as the ultimate molecular or cell structure, as the blood-vessels and nerves."

Overlooking a rather free generalization, which places Dr. Wood in a safe position, in the above quotation, we have an admission which carries us at least a part of the way in the position taken.

Dr. Todd, in his excellent and last work, in his Eleventh Lecture on Pneumonia, says: "The real nature of the organic changes which constitute this state of active conjestion, may be thus explained: Some matter introduced either through the bronchial tubes with the inspired air, or through the blood, irritates a certain portion of the lung; in other words, *disturbs its nervous influence and deranges its nutrition.* The immediate result of this nervous derangement is an increased action of the heart, a dilated and enfeebled state of the contractile walls of the finest capillaries, which offer no resistance to the flow of blood to that part, and allow it to accumulate there in greatly increased quantity. This relaxed state of the capillary wall increases the size of its pores, and allows a freer transudation of liquor sanguinis than takes place in health, and giving rise to those subsequent changes which produce the condition of red hepatization."—p. 237.

I have continued the above quotation farther than needful for my purpose, but the whole is worthy a place anywhere, and especially in the mind of every member of our profession.

M. Claude Bernard says: "It is to the nervous system that we owe both sensibility and voluntary motion, that two-fold source of all our relations to the external world; it presides over all organic functions, and it is my intention to prove to you, that while it is the origin of all the normal phenomena of life, *it is also that of all pathological action.*"

M. Bernard is not a man that would intend to prove a very doubtful problem in physiology, and our position receives strong confirmation in the above quotation from his lectures.

Hastening to a conclusion, some practical inferences must be drawn from the positions taken and supported, perhaps, by arguments not altogether specious, and facts not wholly misplied. If we arrive at the conclusion that nervous irritation is at the foundation of all inflammatory action, it points toward an important indication in treatment, and sets a deffinite limit on the so-called anti-phlogistic measures. It is a maxim in medicine, that he is the safest and wisest practitioner who, knowing well the physiological functions and pathological changes, watches closest, and follows the natural curative process. A foreign substance in the body produces irritation. All the subsequent changes are solely for its removal. The lesson is, remove the cause of irritation, all the steps of inflammation are immediately arrested. Pneumonia, by nature's process, terminates by free sweating, increased flow of urine, and free expectoration. Nature points to her process as an indication whereby this "disturbed nervous influence" may be relieved. Peritonitis terminates by a gradual subsidence of pain and tenderness, cessation of vomiting, and diminished frequency of pulse; sometimes with sweating and free discharge from the bowels or kidneys. What will come so near fulfilling these indications as opium, and what has been proved so efficacious in practice? Here, we may suggest, the remedy acts on the nerves of the part "immediately and bodily," as indicated in the cessation of pain, indicating nervous irritation.

Now this abnormal nutrition, or nervous irritation, to which we give the name of inflammation, is a *depressed state of the vital powers* of the part, and, in extensive inflammation, of the whole system. It is a direct and legitimate effect of the irritant on the nervous system. He who watches for a case of increased vital power or sthenic action in inflammation, will look long. He who practices bloodletting, administers cathartics and emetics, on the principle of sthenic action, will impose upon nature a double burden to bear, and his subsequent measures must be doubly diligent, or his patients will present, not "living witnesses," to his mind, if an observer, of his error. Bloodletting is a remedy so long as it is a recuperator. When it goes beyond unburdening the nervous system, and diminishes nature's material for repair, it becomes pernicious. As already intimated, there is an increase in the waste of tissue. Nutrition is deficient and abnormal, and instead of diminishing the supply the more rational course is to increase the materal. Every step throughout the entire process of inflammation is productive of debility. The increased heat is only a manifestation of rapid oxidization and waste. Pus and Lymph are produced from the blood and tissue, and involve exhaustion. The elimination or absorption of these deposits exhausts the power of the constitution. All these point unmistakably to the principle of treatment. Put no obstruction in the way of the natural process of recovery, nor exhaust nature's powers, but maintain the equilibrium, as far as possible, between waste and repair, and allay irritation by remedies addressed to and acting directly on the part.

